# Tracing Actin Filament Bundles in Three-Dimensional Electron Tomography Density Maps of Hair Cell Stereocilia

**DOI:** 10.3390/molecules23040882

**Published:** 2018-04-11

**Authors:** Salim Sazzed, Junha Song, Julio A. Kovacs, Willy Wriggers, Manfred Auer, Jing He

**Affiliations:** 1 Department of Computer Science, Old Dominion University, Norfolk, VA 23529, USA; ssazz001@odu.edu; 2 Cell and Tissue Imaging, Molecular Biophysics and Integrated Bioimaging Division, Lawrence Berkeley National Laboratory, Berkeley, CA 94720, USA; junhasong@berkeley.edu (J.S.); mauer@lbl.gov (M.A.); 3 Department of Molecular and Cell Biology, University of California, Berkeley, CA 94720, USA; 4 Department of Mechanical and Aerospace Engineering, Old Dominion University, Norfolk, VA 23529, USA; jkovacs@odu.edu (J.A.K.); wriggers@biomachina.org (W.W.)

**Keywords:** cryo-electron tomography, image, density, filament, pattern recognition, segmentation, stereocilia, actin, model building, volumetric model

## Abstract

Cryo-electron tomography (cryo-ET) is a powerful method of visualizing the three-dimensional organization of supramolecular complexes, such as the cytoskeleton, in their native cell and tissue contexts. Due to its minimal electron dose and reconstruction artifacts arising from the missing wedge during data collection, cryo-ET typically results in noisy density maps that display anisotropic XY versus Z resolution. Molecular crowding further exacerbates the challenge of automatically detecting supramolecular complexes, such as the actin bundle in hair cell stereocilia. Stereocilia are pivotal to the mechanoelectrical transduction process in inner ear sensory epithelial hair cells. Given the complexity and dense arrangement of actin bundles, traditional approaches to filament detection and tracing have failed in these cases. In this study, we introduce BundleTrac, an effective method to trace hundreds of filaments in a bundle. A comparison between BundleTrac and manually tracing the actin filaments in a stereocilium showed that BundleTrac accurately built 326 of 330 filaments (98.8%), with an overall cross-distance of 1.3 voxels for the 330 filaments. BundleTrac is an effective semi-automatic modeling approach in which a seed point is provided for each filament and the rest of the filament is computationally identified. We also demonstrate the potential of a denoising method that uses a polynomial regression to address the resolution and high-noise anisotropic environment of the density map.

## 1. Introduction

Our senses of hearing and balance rely on the proper functioning of hair cells [1,2]. Mechanoelectrical transduction occurs at the level of the hair bundle, which comprises individual precision organelles known as stereocilia. Stereocilia are actin bundle-filled membrane protrusions of the apical hair cell surface that develop simultaneously with the maturation of hair cells during the development or regeneration of the inner ear [3]. Research still has not discovered how each stereocilium adopts its defined length and width and how they organize into hair bundles with species- and organ-specific characteristic shapes, though over one hundred proteins have been found to affect hair bundle formation, function, and maintenance [4,5,6,7]. Within individual stereocilia, the precise 3D organization of either a large portion of the actin bundle or the entire bundle has never been determined experimentally; instead, most of our knowledge is derived from projection transmission electron micrograph images of 70–100-nm ultrathin sections viewed in a longitudinal or cross-sectional direction. From such studies, we know that actin filaments generally appear to be spaced ~12 nm apart in a hexagonal pattern in the shaft region, which constitutes the bulk of the stereocilium length. Recently, we conducted a 3D electron tomography of chemically fixed, heavy metal-stained and resin-embedded stereocilia [8], which yielded valuable insight into actin filament packing for both longitudinal sections and cross-sections. However, the analysis was restricted to a small portion of the actin bundle. We have since collected whole-mount cryo-electron tomography data on unstained frozen-hydrated individual stereocilia [9]. The resulting 3D volumes contain over three hundred filaments per stereocilium that must be traced along their entire path. The initial manual tracing has taken weeks for each dataset. Hence, automation in the detection and tracing of actin filaments is highly desirable for prototypic model building. As our long-term goal is the study of the development and regeneration of the hair bundle and its stereocilia 3D macromolecular actin-bundle organization, it is critical that we develop automated approaches to constructing filament models.

Cryo-electron tomography of unstained frozen-hydrated samples is widely seen as the gold standard of the visualization and analysis of the 3D organization of supramolecular complexes in organelles, cells and tissues in their near-native state [10]. However, to avoid damage to the unstained biological specimen, the total allowable electron dose is typically distributed over about 60 images needed for the tilt series data collection. This dose fractionation, and thus the overall dose limitation, unfortunately, results in high-noise 3D reconstructions. Furthermore, limiting the tilt angle and the increased sample thickness at high tilt angles result in anisotropic data, with the Z-direction (along the electron path) showing a resolution decreased by 50% to 66%, depending on the range of the tilt angle and the data quality at high tilt angles. 

**Figure 1:**
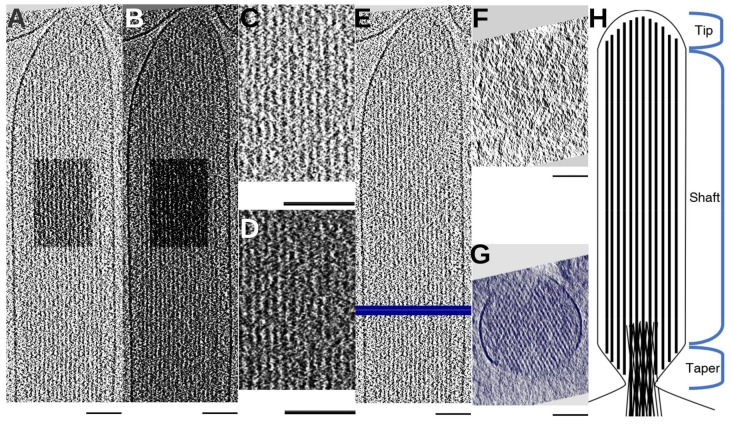
Cryo-ET density map for the shaft of a stereocilium and its membrane-enclosed actin bundle. The longitudinal view of a stereocilium is shown for a slab about 1 nm thick in (**A**) and about 8 nm thick in (**B**). (**C**,**D**) are sub-regions (box) of (**A**,**B**), respectively. (**E**) shows the location of cross-section slices of (**F**) (white line) and (**G**) (blue line) respectively. Cross-sections about 1 nm thick and 30 nm thick are shown in (**F**,**G**), respectively. (**H**) shows the schematic of a stereocilium. All scale bars are 100 nm.

Figure 1 shows the tomographic reconstruction of stereocilia, with actin bundle orientation aligned approximately with the Y (vertical) axis. Figure 1A,B show a ~1-nm single slice and an 8 nm-thick slab of the bundle, respectively. The actin bundle consists of largely parallel actin filaments in close proximity, frequently connected by cross-linking proteins (Figure 1C,D). Figure 1F,G, represent the cross-sectional views of the stereocilium (Figure 1E) of a 1-nm single slice and a slab with a thickness of ~30-nm respectively. The effect of the missing wedge is visible in Figure 1G, with only about one third of the stereocilia membrane being clearly visible and the top and bottom regions of the stereocilia being blurred. Throughout the entire cross-sectional profile, the missing wedge artifact manifests in the elongation of the circular density in the cross-section of an actin filament. The resulting ellipsoids can cause the filaments to merge in the cross-section in the Z-direction (Figure 1G), which makes fully-automated computational tracing approaches rather challenging, as we can no longer rely on the isolation of an individual filamentous structure. When facing multiple filaments in close proximity, template convolution with an atomic model or an abstracted shape, such as a modified cylinder [11], often fails, as the fit is no longer unambiguous due to the difficulty of guarding against jumps between adjacent filaments. In our approach, we exploited the fact that the filaments in the actin bundle are roughly parallel and only change their direction gradually. While it is hard to detect individual filaments in a single cross-sectional slice 1 nm thick (Figure 1F), a longitudinal average of 30 cross-sectional slices (of ~30-nm thickness) significantly enhances the signal and clearly reveals the hexagonal arrangement of the actin filaments (Figure 1G). 

Various automated and semi-automated computational tools have been developed to recognize filamentous objects in medical imaging [12,13,14,15,16]. Locating ridges in a 2D image is a well-studied problem in mathematics and computer vision [17]; however, the 3D equivalent is a newer area of research [18]. Previous work also exists on the segmentation of cytoskeletons in fluorescence images [19] and the tracing of keratin intermediate filaments and neurons in confocal microscope images [20,21,22]. Depending on the nature of the filament network, various theoretical and heuristic approaches have been proposed [23,24,25,26]. In three-dimensional density maps of proteins, helices appear as filamentous objects, and methods have been developed to detect them [27,28,29,30,31,32]. All these methods have in common that they require a low-noise dataset to properly trace the filaments and are thus of limited utility to electron microscopy tomograms.

Fully-automated and semi-automated filament-tracing methods for single tomograms have been proposed and implemented in several different software packages, such as Sculptor and Situs [33], and AMIRA [34]. Sculptor and Situs are non-commercial and freely available, while AMIRA requires a paid license. Rusu et al. developed an automatic method in Sculptor and Situs to trace filaments from a cryo-electron tomogram using cylindrical templates [11]. Weber et al. developed an automated tracing method using modified cylinders that account for the missing wedge artifacts in electron tomography density data [35], and Redemann et al. traced microtubules to build a model of a spindle [36]. Further, segmented filaments can be quantified to understand the organization of a large number of filaments [37]. Unfortunately, these automated approaches for single filaments break down when the close hexagonal spacing of actin filaments and tomography artifacts allow multiple spurious directions to appear as ridges in the 3D volumes.

In this paper, we describe BundleTrac, a method to detect and construct volumetric models of filaments in challenging datasets, such as cryo-tomograms that are characterized by high-noise environments, macromolecular crowding and data anisotropy. This method is designed to utilize the nature of a filamentous bundle, in which filaments do not change abruptly; in other words, changes in filament direction are gradual. We show that it is possible to trace hundreds of filaments over thousands of slices using a semi-automatic approach that relies on an initial set of seed points on a cross-section. We also show that collective tracing and averaging along the estimated filament direction effectively reduces the impact of the noise and artifacts in the tomogram. In a comparison with a manual detection method involving 330 filaments, we found that the filaments detected using BundleTrac have, on average, a close agreement with those detected with the manual method of ~1.3 voxels, well within the thickness of actin filaments (8–10 voxels). In addition, we discuss a denoising method that uses a polynomial kernel regression to create enhanced representations of major local features.

## 2. Results

### 2.1. Longitudinal Average along an Estimated Direction of the Bundle

We designed a simple denoising method based on the filamentous nature of the bundle. The idea was to perform averaging along the filament direction. A critical step in this averaging is determining the overall orientation of the bundle with a two-dimensional (2D) cross-correlation of cross-sections (see Materials and Methods)*.* The cross-sections were sampled at different spots along the bundle to capture the gradual change in the direction of the bundle. Using 2D cross-correlation to identify the axis of a cylindrical object is a quicker alternative to 3D template matching, as it does not involve sampling the translational and rotational space of the object, instead directly calculating the orientation from a trace of the 2D cross-correlation peak shifts. This principle was previously used to identify the axis of VP5 hexons of the capsid shell of the herpes virus [38].

**Figure 2:**
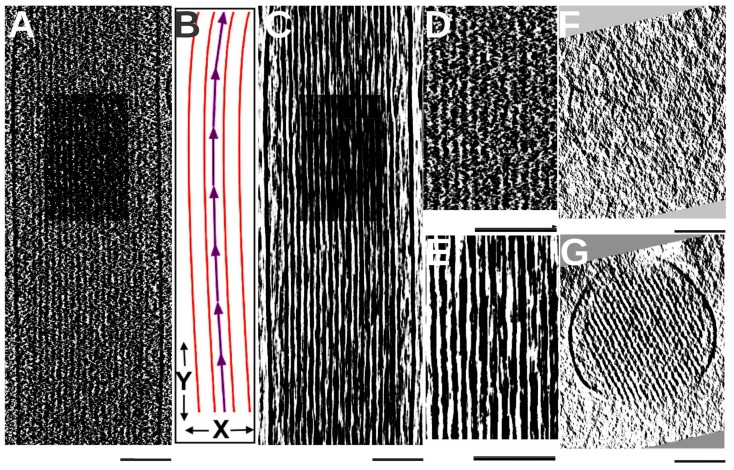
Longitudinal averaging along the direction of the bundle. (**A**) A slab of the stereocilium map ~8 nm thick; (**B**) An illustration of the bundle direction (purple) calculated using 2D cross-correlation of cross-sections; (**C**) An ~8-nm slab of the longitudinally averaged map at the same position as in (**A**); (**D**,**E**) Zoomed-in views of (**A**,**B**), respectively, in the same sub-region (dark box in (**A**,**B**)); (**F**) A ~1-nm cross-section of the original map (**G**) The ~1-nm cross-section of the longitudinally-averaged map at the same position as in (**F**). Note that the cross-sectional view of the averaged map has high clarity; all scale bars represent 100 nm.

Once the orientation of the bundle axis was determined, we calculated the average density along the filament axis. Each voxel was averaged with those within ±15 nm along the direction of the bundle axis. Longitudinal averaging appears to effectively enhance signals along filaments, as the signals are more visible in the longitudinally averaged density slab (Figure 2C,E) than in the original map (Figure 2A,D). This enhancement is clearly visible in a ~1 nm-thick cross-sectional slice but undetectable in the original map (Figure 2F). Density maps displaying a low signal-to-noise ratio make it challenging to directly detect the direction of each individual filament. However, it is possible to detect the direction of a bundle that comprises multiple filaments. Although the direction of the bundle may not be identical to the direction of each individual filament, the former is a close estimate of the latter if the filaments do not suddenly change direction. We used the direction of the bundle as an initial estimate for all filaments in the signal enhancement process.

### 2.2. The Manually Built Model of Actin Filaments

**Figure 3:**
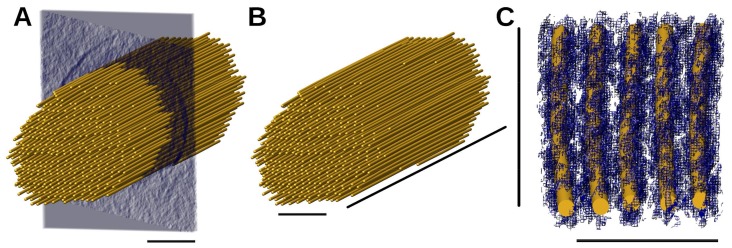
The model of actin filaments, manually constructed as ground-truth. (**A**) Model of stereocilium with cross-sectional slab about 30 nm thick. The scale bar is 100 nm; (**B**) model of stereocilium. The horizontal scale bar is 100 nm, and the diagonal scale bar is 1080 nm; (**C**) local actin filaments of the model superimposed onto the actin densities. The horizontal scale bar is 50 nm, and the vertical scale bar is 540 nm.

After visualizing the 3D tomograms, we focused on a central region of the 3D volume (Figure 3) for the initial manual model building. IMOD [39,40] was used to rotate the tomogram until the actin filaments were aligned with the X-Y plane. This was followed by modelling in UCSF Chimera [41] by manually placing “markers” and “links” (spheres and cylinders) onto the density map of the actin filaments, resulting in an initial, simple ball-and-stick model. The model was then further refined by adjusting the coordinates of the ball-and-stick model using an average of 30 cross-sectional slices. The resulting manually placed ball-and-stick model served as the ground truth for the semi-automated approaches described below.

### 2.3. Filaments Detected Using BundleTrac

Due to the long persistence length of actin filaments, they can be traced by placing markers a certain distance apart along each filament. Without the use of a 3D template, the tracing relies on a 2D convolution of a kernel constructed using seven Gaussian peaks that resemble a local bundle of seven filaments. We applied the seven-peak 2D convolutional optimization method to trace the filaments in the density map of a stereocilium. Volumetric ball-and-stick models for 330 filaments were built from the stereocilium tomogram and compared with those traced manually. Of the 330 filaments, 326 have an average cross-distance less than or equal to three voxels, which is well within the roughly 11–13-voxel distance between two neighboring actin filaments. The average cross-distance of the 330 filaments is 1.3 voxels (Table 1). The cross-distance error measures the average projection distance between two lines over all the points along the lines [42]. The results of the comparison suggest that the two sets of filaments agree with each other very well overall.

**Figure 4:**
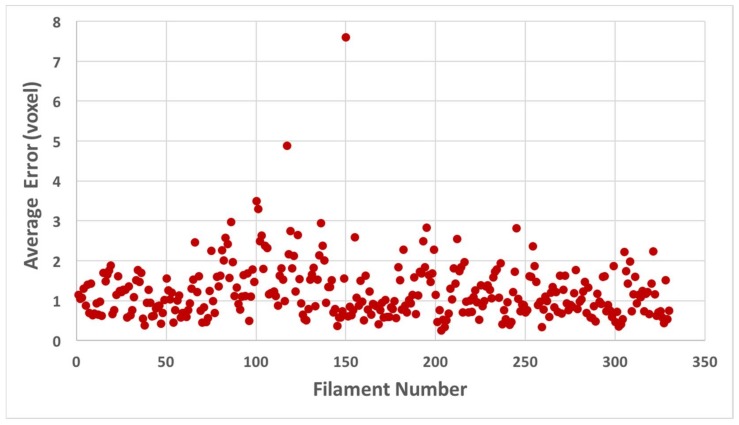
Average cross-distance between a filament detected using BundleTrac and its corresponding manually detected filament for 330 filaments.

The proposed method utilized seed points provided by an expert on a ~30-nm cross-sectional slab. It then traced the entire filament length, constrained by the maximum spacing of filaments (between 11 and 15 nm). The filaments detected using BundleTrac represent locally optimized positions of roughly seven filaments. The underlying assumption, which seems reasonable and is confirmed by manual and semi-automated detection, is that the orientations of the actin filaments only change slowly throughout the 3D volume. Within a 30-nm window along the filament, changes are gradual, so averages provide strong signals. One seed point was provided for each filament. These seeds provided strong constraints on searching the rest of the points that lie along the filaments. Tracing with a convolution of seven Gaussian peaks appears to be effective for 328 of 330 filaments, with two detected filaments significantly deviating from the ground truth with a 5–8-voxel cross-distance (Figure 4). In these two cases, it appears that the filament traces jumped over to the neighboring filament density and were affected by the high degree of local density discontinuities and distortions at certain points along the actin filaments. Additional control may need to be considered in such cases of filament ambiguity.

### 2.4. The Effect of Longitudinal Averaging and Seven-Peak Convolution

**Figure 5:**
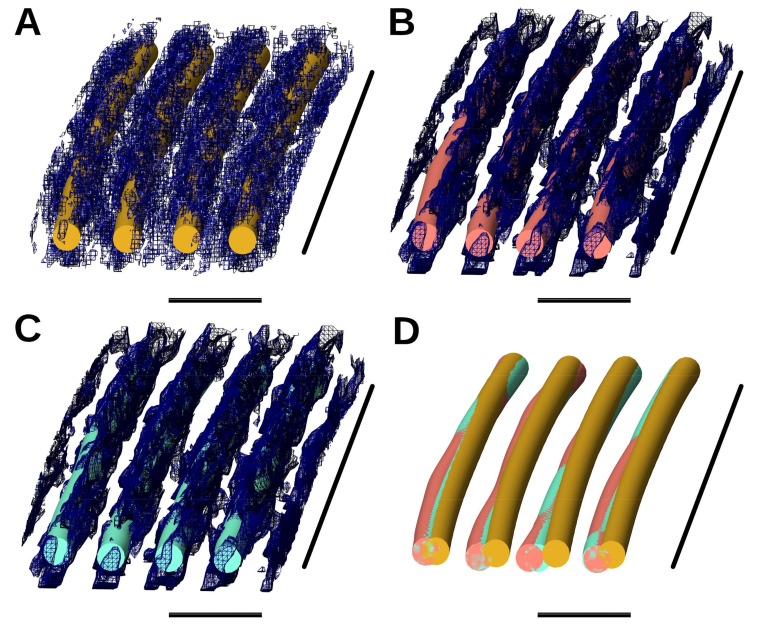
A local view of the filaments built with various implementations. (**A**) Filaments built manually (gold) overlaid with the original map; (**B**) filaments built using the one-peak method (salmon) overlaid with the longitudinally averaged density map; (**C**) filaments built using the seven-peak method (aquamarine) overlaid with the same map as in (**B**); (**D**) superposition of the three sets of filaments in (**A**–**C**). For (**A**–**D**), the horizontal scale bar is 20 nm, and the vertical scale bar is 630 nm.

The major elements of BundleTrac include a longitudinal average and a 2D seven-peak convolution. We analyzed the effectiveness of these two techniques with comparisons among five implementations. For the sake of viewing clarity, we only show here an example of a local region of four single-layer actin filaments (Figure 5). The length of the four filaments is ~630 nm, which is about 1/20 the entire length of the filaments. The longitudinal average appears to increase the signal-to-noise ratio, as the filaments are more obvious in the averaged map (Figure 5B) than in the original map (Figure 5A). In fact, longitudinal averaging reduces the cross-distance error by 14.6%, from 1.5 voxels to 1.3 voxels (Table 1).

Three sets of filament models were compared: those detected manually (gold), those detected using one-peak convolution (salmon) and those detected using seven-peak convolution (aquamarine) (Figure 5). All three sets fit the density well overall, but the filaments detected using seven-peak convolution best align with the manually obtained set (Figure 5). The four filaments for which a portion is shown in Figure 5C have an average cross-distance between 0.36 and 0.39 voxels (3.44 Å–3.67 Å) when seven-peak convolution was applied. The one-peak 2D convolution method traces a filament based on its own density. It is the best method to trace individual filaments if the strength of the signal is sufficient and the artifact of the data is minimized. The seven-peak 2D convolution finds the best superposition of seven hexagonally-arranged Gaussian distributions on the density map at a cross-section of the bundle. It is more robust than the one-peak convolution, as it is less affected by the artifacts and noise in individual filaments. The use of seven-peak convolution reduces the average cross-distance error by 42.3%, from 2.42 voxels for the one-peak convolution to 1.3 voxels for the seven-peak convolution (Table 1). In fact, the most effective technique in our comparison is the seven-peak convolution (46.3%), followed by longitudinal averaging (14.6%). Applying a Gaussian filter to the original map only slightly enhances the accuracy—by 3.3%, from a cross-distance of 1.345 voxels without the filter to 1.3 voxels with it. This is to be expected, because Gaussian filters are isotropic and are not tuned to the artifacts and resolution anisotropy of the experimental data. 

### 2.5. Local Polynomial Regression for Denoising

**Figure 6:**
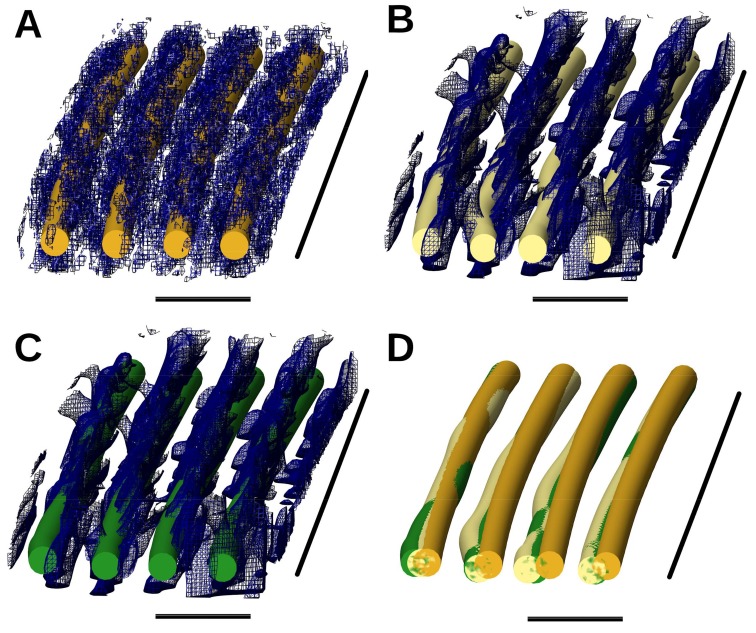
A local view of filaments that were built after applying a polynomial regression for denoising. (**A**) Filaments built manually (gold) overlaid with the original map; (**B**) filaments built using one-peak convolution (khaki) overlaid with the denoised density map created using a polynomial regression; (**C**) filaments built using the seven-peak convolution (green) overlaid with the denoised density map created using a polynomial regression; (**D**) superposition of the three sets of filaments in (**A**–**C**). For (**A**–**D**), the horizontal scale bar is 20 nm, and the vertical scale bar is 630 nm.

BundleTrac employs a simple denoising method involving longitudinal averaging along an estimated filament direction using the bundle. Although it is a fast and effective method of filament detection, specialized denoising methods may generate a more accurate density map for both filament detection and other studies. We explored the idea of using polynomial functions to fit the density locally at each voxel, reassigning the density value as the best-fit polynomial function value at each point. The denoised density map resulting from this local polynomial regression (Figure 6B) appears to display the filaments more obviously than the original density map (Figure 6A). The tracing method of BundleTrac was applied to the denoised map using both one-peak and seven-peak 2D convolution. We observed that one-peak 2D convolution could detect the major trend of the filaments in the preliminary test using a sub-region of the stereocilium tomogram measuring 80 × 1186 × 80 voxels^3^ (Figure 6B). However, the seven-peak convolution generated filaments (Figure 6C,D) that aligned better with the manually detected filaments than those generated by the one-peak convolution. This confirms the effectiveness of the use of the hexagonal bundle in tracing. This exploration demonstrates that the filaments built after the polynomial regression of the denoised map are similar to the manually built filaments, and the more specialized denoising method has the potential to produce a better-quality map. However, the current implementation of polynomial regression method is computationally intensive. Further investigation is needed to develop an efficient denoising method that reduces the artifacts in the tomogram.

BundleTrac is designed to trace filaments that collectively change gradually. Although most filaments experience only a minor change in direction, we observed a more dynamic character of certain filaments when they join a neighboring filament at certain spots along their length. BundleTrac will be less accurate in the case of these “irregular” filaments. In another, larger tomogram of a hair cell stereocilium (500 × 2400 × 500 voxel^3^), we observed multiple such filaments. In this tomogram, BundleTrac found an overall cross-distance of 2.772 voxels for 337 filaments. This stereocilium is roughly twice as long as the first one (348 × 1194 × 483 voxel^3^), in which no “irregular” filaments were observed. In a test of the sub-region of the second stereocilium with none of the “irregular” filaments included, BundleTrac identified 69 filaments with an average cross-distance of 1.519 voxels, which is close to the accuracy obtained from the first stereocilium. Although not currently implemented in BundleTrac, it is possible to detect “irregular” filaments in a post-processing step if they occur in small numbers. In fact, the inter-filament spacing of detected filaments may be used to detect such irregularities in the actin bundle. These spots can be flagged, allowing an expert to determine the reason for the failure, which may have less to do with the algorithm and more to do with the underlying density maps. In such cases, a local (possibly manual) re-tracing may be done by exploring alternative paths.

## 3. Materials and Methods

### 3.1. Cryo-Tomography of Inner Ear Sensory Epithelial Hair Cell Stereocilia

Stereocilia adhering to an electron microscope grid were obtained by gently blotting the mouse’s inner ear utricle sensory epithelium onto the grid lacey-carbon support film [9]. Lacey-coated gold grids with adhering stereocilia were immersion-plunge frozen in liquid ethane at the temperature of liquid nitrogen, and whole-mount intact stereocilia were imaged with a 300-kV FEI Krios microscope using a K2 camera (Gatan Inc., Pleasanton, CA, USA) that was operated in its traditional integrating mode under low-dose conditions. Single axis cryo-tilt series were collected from −60–+60°, with 2° increments at a voxel size of 0.947 nm. Images were aligned and reconstructed using IMOD software [39]. The map was further rotated to align the actin bundle orientation with one of the XYZ coordinate directions. 

### 3.2. Manual Filament Tracing

A visual inspection of eight-slice slabs revealed density maps that showed gaps between adjacent stereocilia, allowing ball-and-stick volumetric models of actin filaments to be placed onto the density map using eight-slice longitudinal slabs. The position of such actin filament models was further refined manually with cross-sectional views.

### 3.3. BundleTrac

**Figure 7:**
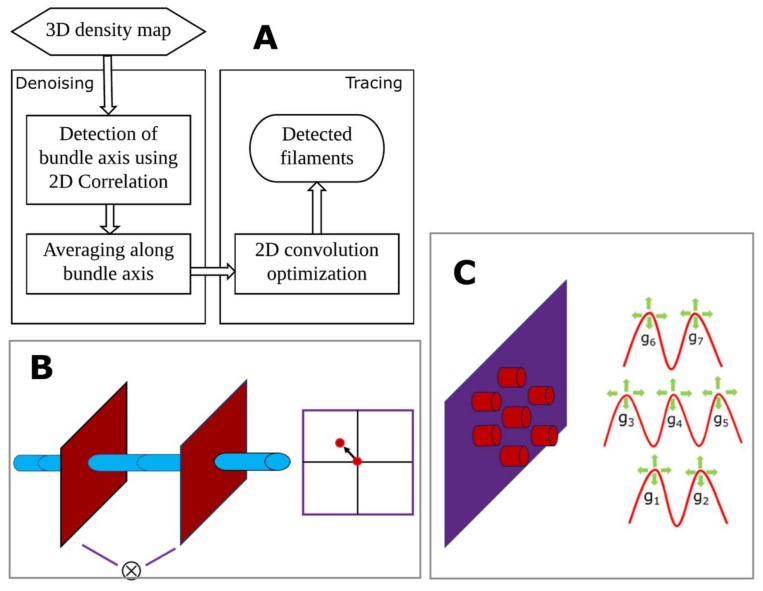
The design of BundleTrac. (**A**) Overall steps; (**B**) detection of the bundle axis using 2D cross-correlation; (**C**) 2D convolutional optimization using seven hexagonally arranged Gaussian peaks.

BundleTrac contains two main steps: (1) the detection of the bundle axis and longitudinal averaging; (2) filament tracing using 2D convolution optimization. As the direction of the bundle is roughly parallel to the Y-axis of the image, we used a series of X-Z slices (approximate cross-sections of the bundle) to calculate the precise direction of the bundle axis. Two cross-sections (the red planes in Figure 7B) were sampled in increments of 55 slices along the Y-axis. This spacing was chosen to capture the gradual change in the direction of the bundle. A 2D cross-correlation was then performed using the two cross-sections to calculate the peak shift. If the bundle is perfectly parallel to the Y-axis, the cross-correlation peak will be at the center of the cross-section, and there will be no peak shift. The degree of the peak shift represents the vector of the bundle at this local region. This process starts from one end of the shaft and extends to the tip region. The overall direction of the bundle comprises a series of vectors (Figure 2B), each generated from 2D cross-correlations, but it is important to note that the use of 2D correlation to detect the bundle axis assumes the architectural rigidity of the bundle. This appears to be the case for the shaft region of stereocilia in the current resolution range. Once the bundle direction was derived, the original 3D density map was averaged along the bundle axis. Specifically, each voxel along the bundle axis was averaged with 15 slices up and down the filament. This denoising step appears to be effective, as the averaging reveals the hexagonal pattern of the filaments (Figure 2G).

To enhance the signal, a kernel was designed by using seven Gaussian functions to mimic a seven-filament neighborhood (Figure 7C). Since the relative distance between two neighboring filaments is slightly different at different locations in the bundle, a local optimization was performed during the 2D convolution to find the best fit. The process started with a set of initial seed points on the first slice of the bundle, which were placed manually using UCSF Chimera [41]. The computational method was then used to identify the remaining markers along each filament. To decide the next marker on each filament, we sampled four downstream cross-sections at varying distances from the current cross-section: 10, 25, 45 and 70 voxels. The best peak value of the seven-peak 2D convolution was selected as the next marker on the filament. We have implemented BundleTrac in a standalone C/C++ program that will be disseminated at the following URL: http://www.cs.odu.edu/~jhe/.

### 3.4. Quantification of the Cross-Distance between Two Sets of Filaments

To assess the accuracy of the method, filaments that were detected computationally were compared with those detected visually. The average cross-distance $D_{f}$ was calculated for each filament (1). The cross-distance measures the projection distance between two lines that may not be straight and may have relative shift [42]. Computationally derived markers are finely interpolated such that, for each visually derived marker, it is possible to find the closest computationally derived point on the corresponding filament. 

(1) $$D_{f}=\overset{n}{\underset{1}{\sum }}P_{i}-Q_{i}$$

$P_{i}$ is a point on the *i*th cross-section (the X-Z plane) of the visually detected filament. $Q_{i}$ is the point on the computationally derived filament that is closest to $P_{i}$. n is the total number of points along a filament. Note that the cross-distance does not reflect the longitudinal distance. The same length was used for a manually detected filament and its corresponding computationally detected filament.

### 3.5. A More Specialized Denoising Method

The denoising approach described above is very simple, which increases the efficiency of the procedure. In order to evaluate the dependence of the model’s predictive performance on the denoising, we developed a more specialized method (the denoising of tomograms is a relatively mature field; for a more comprehensive review of the methods used by us and others, see Starosolski et al. [43]). 

Our more specialized method is an adaptation of a type of regression called a local polynomial kernel regression, which is non-parametric (i.e., model-free) [44]. The degree of the polynomial that we used is two, which means that to each point of the map, we fit a quadratic polynomial of three variables (*x*, *y*, *z*), the general expression of which is:

(2) $$f\left(x,y,z\right)=a_{11}x^{2}+a_{12}xy+a_{13}xz+a_{22}y^{2}+a_{23}yz+a_{33}z^{2}+b_{1}x+b_{2}y+b_{3}z+c$$

The word “kernel” in the name of the method refers to the weights that map the values that surround the particular point and will be given during the fitting. If we call this kernel *W*, then the fitting criterion at a point *p* is the minimization of the following expression on the coefficients of the polynomial shown above:

(3) $$\sum_{q}{\left(f\left(q\right)-\Phi \left(q\right)\right)}^{2}\;W\left(q-p\right)\to \;\;\;\;\;\min$$

where Φ is the given map and the summation is taken over the entire map. Once the coefficients are determined, the value of the denoised map at point *p* is defined as *f*(*p*).

For the problem we address in this paper, the “weight kernel” *W* is constructed as the convolution of two other kernels:

(4) W=S∗R

where *S* is the “shape kernel” and *R* is the “resolution kernel.” The shape kernel encapsulates the basic information we have about what we expect to see in the map, specifically the filaments. Thus, this kernel is treated as a Gaussian function, with a shape that is elongated in the direction of the filaments and a diameter in the orthogonal plane that is the expected thickness of the filaments.

The resolution kernel *R* is a simplified form of the point-spread function of the imaging system. Due to the “missing wedge” artifacts, the exact shape of this point-spread function is complicated, so we instead use a Gaussian approximation of its central peak, which (again due to the missing wedge) is not isotropic, but elongated in the Z direction. The estimated width of this Gaussian approximation along Z is taken to be twice its value in the XY plane. 

Since the kernels *S* and *R* are both Gaussian, their convolution will be Gaussian as well:

(5) $$W\left(x\right)=\exp\left(-\frac{1}{2}\;x\cdot H^{-1}\cdot x^{T}\right)$$

where $H=H_{S}+H_{R}$ is the sum of the covariance matrices of the two kernels.

## 4. Conclusions

The long shaft region of stereocilia consists of hundreds of mostly parallel actin filaments organized as a bundle. Although automatically tracing these filaments remains a goal, it is complicated by the limited resolution of cryo-ET datasets, the high level of noise and the dense 3D organization of the actin bundle. This study presents a semi-automated filament tracing method, BundleTrac, that is specifically designed for the detection of filaments in a bundle. An effective image processing method often utilizes prior knowledge of the characteristics of the relevant object. BundleTrac utilizes the characteristics of a bundle in two ways. It quickly calculates the bundle axis via a method involving 2D cross-correlation on sampled cross-sections. The bundle axis is then used to denoise the map through longitudinal averaging. The tracing of the filaments also utilizes the nature of a bundle. In this study, we presented a method of tracing a filament using a seven-peak 2D convolution optimization that utilizes the hexagonal packing of filaments.

This method is effective at tracing filaments in a bundle. In a comparison with manually traced filaments of a stereocilium, BundleTrac detected 326 of the 330 filaments with an average cross-distance of less than three voxels, well within the 11–13 voxels that exist between neighboring filaments. The overall cross-distance of the 330 filaments is 1.3 voxels. The detected filaments align well with the manually built filaments. In this paper, we also introduced a polynomial regression method for denoising density maps. The potential of this method was demonstrated in a preliminary test, in which the filament models detected from the denoised map aligned well with the manually detected filaments. 

The use of manual seeding is generally acknowledged to be a reasonable strategy in tracing actin filaments [11,37] and microtubules [35]. BundleTrac relies on the assumption that the filaments in the bundle collectively change in a gradual manner. In future work, we will extend this approach to the so-called “taper” region of the stereocilium, in which the paths of individual actin filaments must be followed individually but the criterion of gradual changes in orientation still applies. However, such analysis, which will generalize this method, requires developments beyond the scope of the work presented here. 

## Figures and Tables

**Table 1:** The effect of longitudinal averaging, isotropic Gaussian filter, seven-peak 2D convolution, and one-peak 2D convolution on overall accuracy.

Row	Implementation	Avg_L ^a^	Gauss ^b^	7 Peaks ^c^	1 Peak ^d^	AvgError ^e^
1	Trace_L_G_7	✔	✔	✔	✖	1.300
2	Trace_L_7	✔	✖	✔	✖	1.345
3	Trace_G_7	✖	✔	✔	✖	1.523
4	Trace_L_1	✔	✖	✖	✔	2.157
5	Trace_L_G_1	✔	✔	✖	✔	2.420
Improvement in the seven-peak convolution ^f^						46.28%
Improvement in the longitudinal average ^g^						14.62%

^a^ Longitudinal average along the direction of the bundle; ^b^ isotropic Gaussian filter applied to the original density map; 2D convolution using ^c^ seven Gaussian peaks and ^d^ one Gaussian peak; ^e^ average cross-distance (in voxels) between the manually and computationally detected 330 filaments. A check mark indicates that a technique in the column was applied. For example, 1.300 voxels was the average cross-distance when the longitudinal average, isotropic Gaussian filter and seven-peak convolution were applied. ^f^ Improvement = difference in error between row 5 and row 1/error in row 5; ^g^ improvement = difference in error between row 3 and row 1/error in row 3.
